# Bifunctionality from Synergy: CoP Nanoparticles Embedded in Amorphous CoOx Nanoplates with Heterostructures for Highly Efficient Water Electrolysis

**DOI:** 10.1002/advs.201800514

**Published:** 2018-07-13

**Authors:** Jie Yu, Yijun Zhong, Xinhao Wu, Jaka Sunarso, Meng Ni, Wei Zhou, Zongping Shao

**Affiliations:** ^1^ Jiangsu National Synergetic Innovation Center for Advanced Materials (SICAM) State Key Laboratory of Materials‐Oriented Chemical Engineering College of Chemical Engineering Nanjing Tech University No. 5 Xin Mofan Road Nanjing 210009 P. R. China; ^2^ Department of Chemical Engineering Curtin University Perth Western Australia 6845 Australia; ^3^ Research Centre for Sustainable Technologies Faculty of Engineering, Computing and Science Swinburne University of Technology Jalan Simpang Tiga Kuching 93350 Sarawak Malaysia; ^4^ Building Energy Research Group Department of Building and Real Estate The Hong Kong Polytechnic University Hung Hom Kowloon 999077 Hong Kong China

**Keywords:** amorphous cobalt oxide nanoplates, crystalline cobalt phosphide nanoclusters, electronic interactions, heterostructures, nanointerfaces, water electrolysis

## Abstract

Hydrogen production from renewable electricity relies upon the development of an efficient alkaline water electrolysis device and, ultimately, upon the availability of low cost and stable electrocatalysts that can promote oxygen evolution reaction (OER) and hydrogen evolution reaction (HER). Normally, different electrocatalysts are applied for HER and OER because of their different reaction intermediates and mechanisms. Here, the synthesis of a heterostructured CoP@a‐CoOx plate, which constitutes the embedded crystalline cobalt phosphide (CoP) nanoclusters and amorphous cobalt oxides (CoOx) nanoplates matrix, via a combined solvothermal and low temperature phosphidation route is reported. Due to the presence of synergistic effect between CoP nanoclusters and amorphous CoOx nanoplates in the catalyst, created from the strong nanointerfaces electronic interactions between CoP and CoOx phases in its heterostructure, this composite displays very high OER activity in addition to favorable HER activity that is comparable to the performance of the IrO_2_ OER benchmark and approached that of the Pt/C HER benchmark. More importantly, an efficient and stable alkaline water electrolysis operation is achieved using CoP@a‐CoOx plate as both cathode and anode as evidenced by the obtainment of a relatively low potential of 1.660 V at a 10 mA cm^−2^ current density and its marginal increase above 1.660 V over 30 h continuous operation.

## 1 Introduction

Hydrogen, a zero carbon emission energy carrier, is considered as one of the most environmentally benign and renewable alternatives to fossil fuels.^1^ However, hydrocarbon reforming is currently the main pathway to produce hydrogen, which is associated with high fossil fuel consumption and significant amount of CO_2_ release.[[qv: 1e]] To this end, alkaline water electrolysis, one of the most facile processes for hydrogen production, serves as an attractive technology route to produce high‐purity hydrogen from electricity that comes from renewable energy resources.^2^ It essentially combines two half‐cell reactions, i.e., hydrogen evolution reaction (HER, 2H_2_O + 2e^−^ → H_2_ + 2OH^−^) and oxygen evolution reaction (OER, 4OH^−^ → O_2_ + 2H_2_O + 4e^−^).^3^ Currently, one of the main challenges toward widespread use of water electrolysis is how to reduce the energy consumption and the cost, primarily pursued via the research and development of alkaline water electrolysis technology on its several aspects, i.e., the electrodes (containing electrocatalysts), the electrolytes, the ionic transport, and the bubble formation.[[qv: 3d]] Electrocatalysts, in particular, can facilitate charge transfer or chemical reaction; providing the reduced activation energy of the reaction as reflected by the reduced overpotential of either or both of the two half reactions.[[qv: 3d,4]] Thus, the design and synthesis of highly efficient electrocatalysts for the HER and OER under an alkaline condition is critical to decrease the overall energy losses in alkaline water electrolysis.^3^, ^4^ To this end, low cost yet highly active HER and OER catalysts have been sought upon from different alternative family of materials such as transition metal oxides, (oxy)hydroxides, phosphides, carbides, nitrides, sulfides, their hybrids, pure carbon materials, and carbon‐based hybrids.[[qv: 2b,3–5]] Among them, cobalt phosphide in particular displayed high catalytic potential for water electrolysis; the performance of which has been further enhanced via morphology control, coupling with conductive carbon, and doping with other metals such as Fe, Mn, and Ni.^6^ Despite the significant progress, the existing works on these alternative materials have nevertheless been performed exclusively in an acidic electrolyte for HER and in an alkaline electrolyte for OER,[[qv: 4b,6a]] which do not reflect the practical water electrolysis scenario where the coupling of HER and OER catalysts should occur in a single electrolyte.

Developing a bifunctional catalyst that can simultaneously provide high HER and OER activities in a single electrolyte thus represents a more sensible approach to enable the electrolyzer design simplification and cost reduction. Heterostructuring approach, which features strong coupled interface between the different active components to enable synergistic effects and enhanced electron transfer rate at the interfaces, has been applied to develop competitive bifunctional catalysts.[[qv: 4b,5a,e,f,7]] Three years ago, Dai and co‐workers[[qv: 5a]] found the serendipitous formation of nanoscale nickel oxide/nickel heterostructures on carbon nanotube sidewalls that enables very high HER activity on a level comparable to Pt. Hu and co‐workers[[qv: 5f]] also reported high electrocatalytic water oxidation performance of the Janus catalyst core‐shell structured Ni_2_P/NiOx nanoparticles in a 1 m KOH electrolyte. These reports allude towards the possible enhancement of HER and OER activities via the formation of intimately coupled interfaces between the HER and OER active components. Recently, Xue et al.[[qv: 4b]] synthesized Co/CoP nanoparticles that show simultaneously high HER and OER performances. They attributed these performances to the Janus structure of Co/CoP nanoparticles and the resultant Mott–Schottky interactions between these two nanoparticles.

In this work, we report the facile synthesis and characterization of cobalt phosphide (CoP) nanoparticles embedded in amorphous cobalt oxides (CoOx) nanoplates with heterojunction‐like structure (CoP@a‐CoOx plate), which exhibited simultaneously high HER and OER activities in an alkaline (1 m KOH) solution. Notably, its OER activity slightly surpassed that of IrO_2_ while its HER activity approached that of Pt/C. The CoP@a‐CoOx plate formed following the combined solvothermal process and low temperature phosphidation route. We attributed its outstanding bifunctional catalytic activity to the synergy created from the nanointerfaces strong electronic interactions between the CoP and the amorphous CoOx in its heterostructure. More importantly, such excellent bifunctional performance also translated to a high water‐splitting performance as evidenced by a relatively low water splitting potential of ≈1.660 V at a 10 mA cm^−2^ current density.

## 2 Results and Discussion

**Scheme 1:**
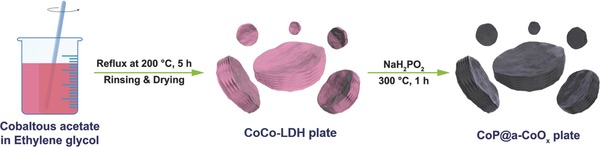
Synthesis procedure of the CoP@a‐CoOx plate.

The synthesis procedure of CoP@a‐CoOx plate is depicted in **Scheme** **1** (see the Experimental Section for the details). First, CoCo layered double hydroxides precursors (CoCo‐LDH plate) were prepared via a one‐pot solvothermal route by refluxing cobaltous acetate in ethylene glycol (EG) media at 200 °C. Subsequently, the resultant CoCo‐LDH plate was phosphidated at 300 °C using phosphine vapor from sodium hypophosphite. Following such phosphidation, the color of the CoCo‐LDH plate changed from pink to black, suggesting the formation of phosphides in the final CoP@a‐CoOx plate.

**Figure 1:**
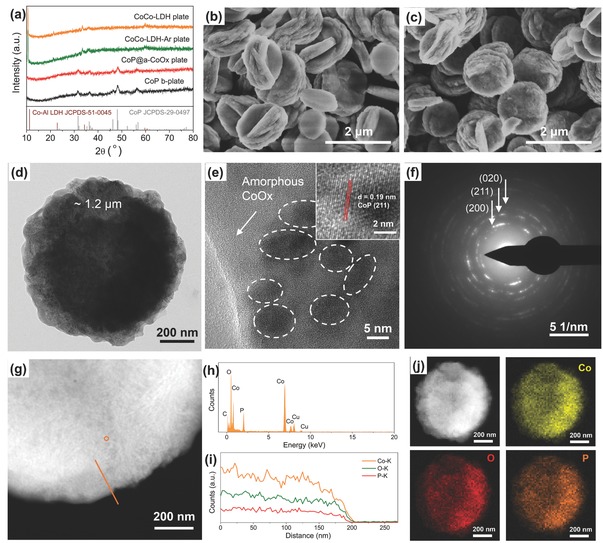
a) Powder XRD patterns of the CoCo‐LDH plate, the CoCo‐LDH‐Ar plate, the CoP@a‐CoOx plate, and the CoP b‐plate samples. b,c) SEM images of the CoCo‐LDH plate and the CoP@a‐CoOx plate, respectively, that contain ≈1.2 µm diameter round platelets. d) TEM image, e) HR‐TEM image, and f) SAED pattern of the CoP@a‐CoOx plate. g) HAADF‐STEM image of the CoP@a‐CoOx plate with an orange line showing the line scanning path, i) the corresponding line‐scanning profile, h) EDX spectrum of the CoP@a‐CoOx plate measured at position P in (g). j) HAADF‐STEM image of the CoP@a‐CoOx plate and the corresponding element mapping images displaying the uniform spatial distribution of Co (yellow), O (red), and P (orange).

Powder X‐ray diffraction (XRD) measurements were performed to evaluate the phase structure of the samples; the results of which were shown in **Figure** **1**a. All diffraction peaks of the CoCo‐LDH plate, which are relatively weak in intensity, can be indexed according to the characteristic peaks of the hydrotalcite like materials (Co_6_Al_2_CO_3_ (OH)_16_·4H_2_O, CoAl‐LDH, JCPDS No. 51‐0045) despite the slight shift in position; indicating the successful preparation of the CoCo layered double hydroxide (CoCo‐LDH) (Figure 1a – CoCo‐LDH plate), as reported in several previous articles.^8^ The CoCo‐LDH‐Ar plate, i.e., the CoCo‐LDH plate subjected to argon (Ar) atmosphere in the absence of sodium hypophosphite, retained an analogous powder XRD pattern to CoCo‐LDH plate; indicating the retainment of the CoCo‐LDH phase after such treatment (Figure 1a – CoCo‐LDH‐Ar plate). Following the phosphidation reaction at 300 °C nonetheless, six characteristic peaks at 2θ of 31.6°, 36.5°, 46.2°, 48.2°, 52.3°, and 56.7°, which represented the (011), (111), (112), (211), (103), and (301) planes of the crystalline CoP phase (JCPDS No. 29‐0497), respectively, appeared while the previously observed CoCo‐LDH plate peaks disappeared (Figure 1a – CoP@a‐CoOx plate). This signified the phase transition from CoCo‐LDH to CoP after phosphidation. As a baseline comparison, acid etching was performed on a portion of CoP@a‐CoOx plate sample, the product of which we denoted as CoP b‐plate (Figure 1a – CoP b‐plate). CoP b‐plate displayed an analogous pattern to CoP@a‐CoOx plate.

The morphology of the samples was characterized using scanning electron microscopy (SEM) and transmission electron microscopy (TEM). SEM images of the CoCo‐LDH plate and the CoP@a‐CoOx plate (Figure 1b,c, respectively) revealed the existence of ≈1.2 µm diameter round platelets for the pink‐colored CoCo‐LDH precursors; the shape and size of which were preserved following the phosphidation reaction. The surface of the CoP@a‐CoOx plate however looked rougher than that of the CoCo‐LDH plate. Further acid etching of the CoP@a‐CoOx plate appeared to disintegrate the platelets (Figure S1, Supporting Information). This indicated that the constituents of the CoP@a‐CoOx plate matrix were unlikely to be entirely crystalline but rather a mixture of crystalline CoP and amorphous acid‐soluble cobalt oxides (CoOx). The TEM image of the CoP@a‐CoOx plate (Figure 1d) displayed an individual round platelet. Upon focusing the electron beam into the edge portion of such individual platelet, we detected numerous clusters (dark spots as marked by white circles) embedded in an amorphous phase (Figure 1e). These dark spots featured a distinct lattice fringe with a regular spacing of 0.19 nm (inset of Figure 1e), which matched the (211) plane of the CoP phase and indicated the CoP origin of such nanoclusters. The interspersion of crystalline clusters in amorphous matrix suggested their intimate contacts and interactions. Selected area electron diffraction (SAED) pattern additionally showed the formation of spots representative of the crystalline CoP and diffuse rings characteristic of the amorphous CoOx (Figure 1f); supporting our aforementioned hypothesis on the amorphous phase constituent of the plate. The atomic ratio of Co, O, and P for CoP@a‐CoOx determined from the energy dispersive X‐ray (EDX) analysis was ≈1:0.75:0.33 (Figure 1g;h). The presence of relatively large amount of O and the large deviation of Co:P ratiofrom 1:1, again, corroborated the existence of CoOx. High‐angle annular dark‐field scanning transmission electron microscopy (HAADF‐STEM)‐EDX line scan and mapping results (Figure 1g,i,j) additionally revealed the uniform spatial distribution of Co, O, and P over the marked detection range on the CoP@a‐CoOx plate sample. To further reveal the amorphous constituents in the final material, the qualitative determination of gas components (H_2_O) of the CoP@a‐CoOx plate and CoCo‐LDH during the thermal decomposition process was obtained using thermogravimetry‐mass spectrometry experiment. As shown in Figure S2 of the Supporting Information, a strong peak that came from H_2_O was observed for the CoCo‐LDH plate, which was absent for the CoP@a‐CoOx plate; indicating that the amorphous components were oxides. Using nitrogen (N_2_) adsorption–desorption isotherms (Figure S3; Supporting Information), we calculated the Brunauer–Emmett–Teller (BET) surface areas of CoCo‐LDH plate, CoP@a‐CoOx plate, CoCo‐LDH‐Ar plate, and CoP b‐plate, which were 61.1, 18.2, 37.0, and 32.9 m^2^ g^−1^, respectively (Table S1, Supporting Information).

**Figure 2:**
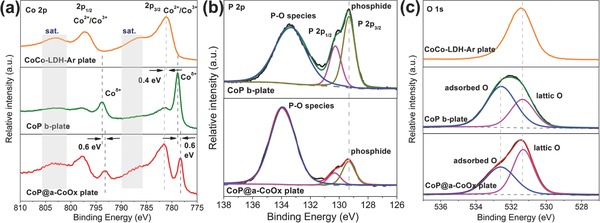
High‐resolution XPS spectra of a) Co 2p, b) P 2p, and c) O 1s for different samples. In (a) and (c) from top to bottom: CoCo‐LDH‐Ar plate, CoP b‐plate, and CoP@a‐CoOx plate. In b) from top to bottom: CoP b‐plate and CoP@a‐CoOx plate.

X‐ray photoelectron spectroscopy (XPS) measurements were performed to evaluate the surface chemical composition and the oxidation state change after phosphidation. High‐resolution Co 2p, P 2p, and O 1s spectra of CoCo‐LDH‐Ar plate, CoP@a‐CoOx plate, and CoP b‐plate were shown in **Figure** **2**a–c, respectively. Note that P 2p spectrum for CoCo‐LDH‐Ar plate is absent in Figure 2b since it does not contain P. There were two regions in Co 2p spectrum, i.e., Co 2p3/2 region at low binding energy range and Co 2p1/2 region at high binding energy range, which exhibited a one‐to‐one correspondence between each other (Figure 2a). In CoCo‐LDH‐Ar plate case, two main peaks that appeared in Co 2p3/2 region, i.e., at 781.1 and 786.6 eV can be attributed to the oxidized Co^2+^/Co^3+^ in Co(OH)_2_/CoOOH and the corresponding shake‐up satellite peak, respectively (Figure 2a – top panel). After phosphidation, an additional, intense Co 2p3/2 peak at 778.3 eV came out, which can be assigned to Co—P bonding and signified the presence of Co^δ+^ (Figure 2a – bottom panel). It is worth noting that this peak position lied in very close proximity to the reported position of the metallic Co (δ = 0) at 778.1 eV and deviated slightly from 778.9 eV position for CoP widely observed in several previous reports.[[qv: 3a,4b,6a]] CoP b‐plate, by contrast, displayed Co^δ+^ peak at 778.9 eV (Figure 2a – center panel). To this end, the shifting of Co^δ+^ peak in the CoP@a‐CoOx plate case likely indicated the modification in the electronic structure due to the two strongly coupled phases (Figure 2a – center and bottom panels). Moreover, relative to the CoCo‐LDH‐Ar plate, the Co^2+^/Co^3+^ peaks for the CoP@a‐CoOx plate and the CoP b‐plate were shifted by ≈0.4 eV to higher binding energy positions (Figure 2a – all panels); most probably due to the higher Co^2+^ content in these two samples. Closer comparison on the Co^2+^/Co^3+^ peaks in the CoP b‐plate and CoP@a‐CoOx plate spectra also revealed the reduction in the intensity of this peak following acid etching which confirmed the presence of the amorphous CoOx phase (Figure 2a – center and bottom panels). The remaining weak Co^2+^/Co^3+^ peak at 781.5 eV in CoP b‐plate case likely came from the cobalt ions in the phosphate; generated by the surface oxidation of phosphide.[[qv: 4d,6a,c,d]] Moving onto P 2p spectra next, three deconvoluted peaks could be observed with that the two peaks at 129.3 and 130.3 eV for P 2p_3/2_ and P 2p_1/2_, respectively, signified phosphide, and another one at a higher binding energy of ≈134 eV indicated phosphate‐like P species (from PO_4_^3−^ or P_2_O_5_) that came mainly from the oxidized surface of metal phosphides (Figure 2b).[[qv: 4d,6a,c,d]] Upon comparing P 2p spectrum of the CoP b‐plate to that of the CoP@a‐CoOx plate, it became clear that the intensity of phosphate‐like P species relative to phosphide species for the latter was significantly stronger than the former (Figure 2b – bottom and top panels). Our speculation attributed this to the presence of amorphous CoOx component that might attenuate the XPS signal collection from CoP component. High resolution O 1s spectrum for CoCo‐LDH‐Ar plate displayed a distinct characteristic peak of lattice oxygen (Figure 2c – top panel). In the CoP@a‐CoOx plate case nonetheless, its O 1s peak clearly showed the sign of overlapped peaks, which could be deconvoluted into lattice oxygen and adsorbed O species (Figure 2c – bottom panel). This observation served as another supporting evidence of the amorphous CoOx species content on the CoP@a‐CoOx plate sample. Despite its defined single peak shape for the CoP b‐plate, the O 1s peak can be deconvoluted into adsorbed O species and a small amount of lattice oxygen, and the lattice oxygen raised from the inevitable surface oxidation, in well agreement with Co 2p and P 2p spectra analysis (Figure 2c – center panel). Overall, our XPS observation, in particular the shifting of Co 2p binding energy position for the CoP@a‐CoOx plate implied the change in the electronic structure from the strong electronic interaction that might enhance the electrocatalytic activity of the CoP@a‐CoOx plate as demonstrated in other composite systems.[[qv: 4b,5a,e,f,7]]

**Figure 3:**
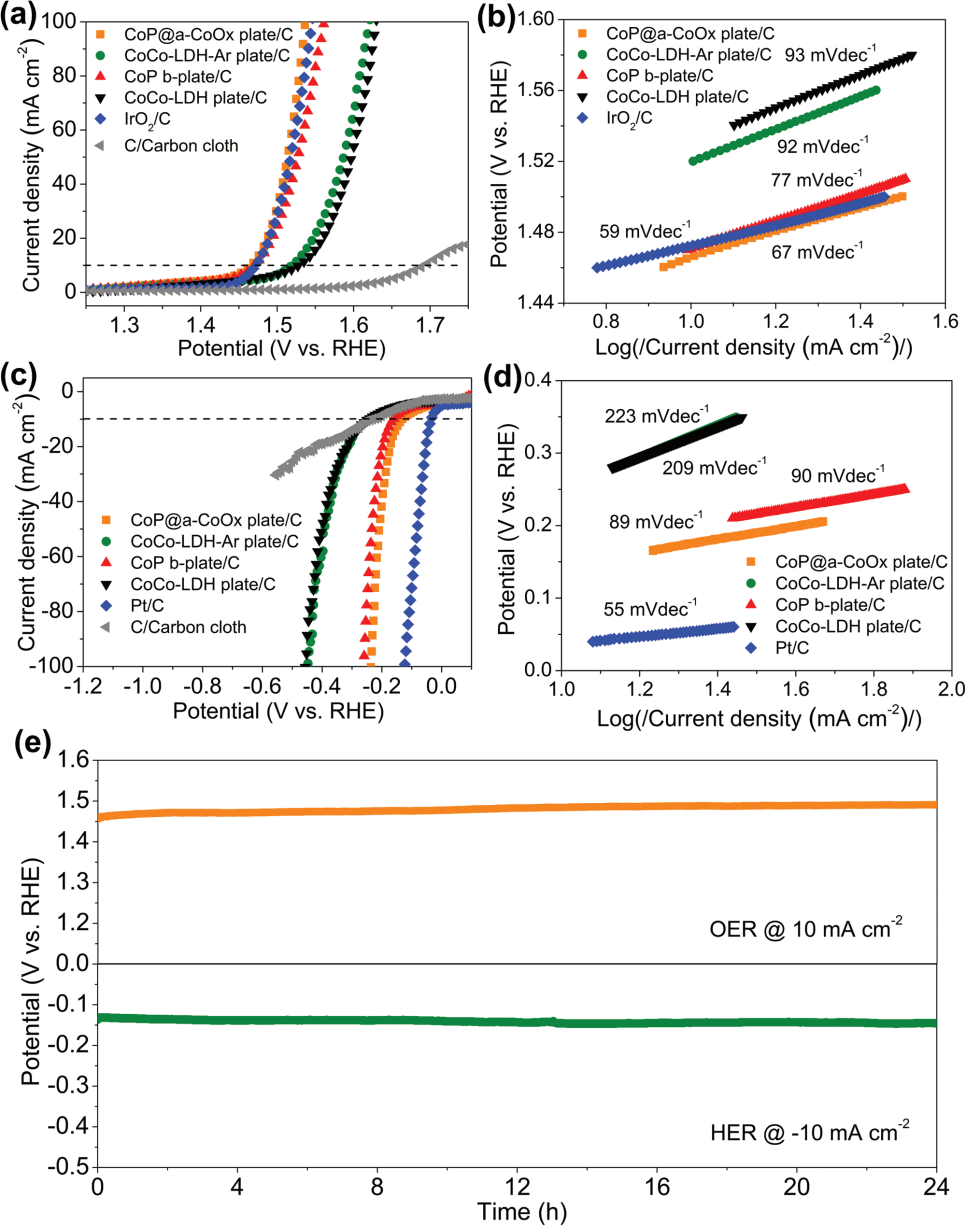
a) OER polarization curves and b) the corresponding Tafel plots of the CoP@a‐CoOx plate/C, the CoCo‐LDH‐Ar plate/C, the CoP b‐plate/C, the CoCo‐LDH plate/C, and the commercial IrO_2_/C loaded on the carbon cloth substrate in an O_2_‐saturated 1 m KOH solution; obtained using a 5 mV s^−1^ scan rate. c) HER polarization curves, and d) the corresponding Tafel plots of the CoP@a‐CoOx plate/C, the CoCo‐LDH‐Ar plate/C, the CoP b‐plate/C, the CoCo‐LDH plate/C, and the commercial Pt/C loaded on the carbon cloth substrate in an Ar‐saturated 1 m KOH solution, obtained using a 5 mV s^−1^ scan rate. e) Chronopotentiometric (potential versus time) plots for the CoP@a‐CoOx plate under constant current densities of 10 mA cm^−2^ and −10 mA cm^−2^ for OER and HER, respectively.

We subsequently evaluated the OER and HER activities of the CoP@a‐CoOx plate, the CoCo‐LDH‐Ar plate, and the CoP b‐plate by recording the respective polarization curves using linear sweep voltammetry (LSV) in an O_2_ or Ar‐saturated 1 m KOH electrolyte (O_2_ for OER test and Ar for HER test). As a baseline comparison, we also recorded the relevant polarization curves from the commercial IrO_2_ or Pt/C. All catalyst samples used were mixed with the conductive carbon (C) additive to remove the electronic conductivity limitation and increase dispersibility of the sample in the solvent media during catalyst ink preparation. Such carbon component additive on carbon cloth made negligible contribution to OER signal below 1.6 V versus reversible hydrogen electrode (RHE) (**Figure** **3**a). The CoP@a‐CoOx plate showed very high OER activity, i.e., an overpotential of 0.232 V at a 10 mA cm^−2^ current density (η_10_), which surpassed those of the CoP b‐plate and the CoCo‐LDH‐Ar plate (with η_10_ = 0.242 and 0.293 V, respectively); highlighting the synergistic effect of the two strongly coupled phases. Notably, the OER activity of the CoP@a‐CoOx plate even slightly outperformed that of the IrO_2_ catalyst benchmark (with η_10_ = 0.243 V) and significantly exceeded those of numerous leading nonprecious metal‐based OER catalysts in an alkaline electrolyte (Table S2, Supporting Information). Tafel plots can then be drawn from the polarization curves to reveal the OER kinetics behavior (Figure 3b). The Tafel slope of the CoP@a‐CoOx plate was 67 mV dec^−1^, which was lower than those of the CoCo‐LDH‐Ar plate (92 mV dec^−1^) and CoP b‐plate (77 mV dec^−1^) catalysts; indicating its much faster reaction kinetics. It was nonetheless larger than that of IrO_2_ (59 mV dec^−1^). The OER kinetics of the CoCo‐LDH‐Ar plate and the CoCo‐LDH plate appeared to have the same kinetics mechanism given their identical Tafel slopes.

The CoP@a‐CoOx plate also displayed high HER activity, i.e., an overpotential of 0.132 V at a 10 mA cm^−2^ current density (η_10_), which was about 110 and 30 mV lower than those of the CoCo‐LDH‐Ar plate and the CoP b‐plate (Figure 3c). We confirmed that HER signal contribution from conductive carbon on carbon cloth to all tested catalyst samples is minor above −0.5 V versus RHE (Figure 3c). Although Pt/C clearly showed superior HER activity than the CoP@a‐CoOx plate, detailed comparison with the nonprecious metal‐based HER catalysts benchmarks in an alkaline electrolyte highlighted the comparable performance of the CoP@a‐CoOx plate relative to these benchmarks (Table S3, Supporting Information). In addition, the fact that the CoP@a‐CoOx plate exhibited a lower Tafel slope of 89 mV dec^−1^ relative to those of the CoCo‐LDH‐Ar plate and the CoP b‐plate (90 and 223 mV dec^−1^, respectively) demonstrated a more favorable HER kinetics for the former (Figure 3d). To retain a more accurate and objective comparison, the overpotentials at the current densities of 10, 50, and 100 mA cm^−2^ for both OER and HER of the different catalysts, respectively, were presented in the form of scatter diagram with error bars (Figure S4, Supporting Information). The superior OER performance in addition to the high HER performance demonstrated by the CoP@a‐CoOx plate catalyst verified its outstanding bifunctional catalytic performance that is required to achieve economic water electrolysis.

To assess the impact of the CoP content in the heterostructure on the electrocatalytic performance of HER and OER, the ratio of the crystalline CoP nanoclusters to the CoP@a‐CoOx heterostructure hybrid was optimized in control experiments by varying the weight ratios of NaH_2_PO_2_ and CoCo‐LDH precursors. Two other catalysts with the different weight ratios of NaH_2_PO_2_ and CoCo‐LDH precursors (5:1 and 20:1) were prepared and are denoted as CoP@a‐CoOx‐5 plate and CoP@a‐CoOx‐20 plate, respectively. Figure S5 (Supporting Information) displayed the powder XRD patterns of these samples with different CoP contents. As the more NaH_2_PO_2_ was involved, the diffraction peaks assigned to the CoP phase became much stronger. TEM images of the three samples revealed almost identical micromorphology (Figure 1; Figure S6, Supporting Information). These samples also had identical BET surface areas (Table S1, Supporting Information). Moving onto XPS analysis (Figure S7, Supporting Information), all of them showed the analogous high‐resolution Co 2p and P 2p spectra. However, the intensity of Co^δ+^ species (Co—P) relative to Co^2+^/Co^3+^ (Co—O) in the high‐resolution Co 2p spectrum or phosphide species relative to phosphate‐like P species in the high‐resolution P 2p spectrum for the CoP@a‐CoOx‐5 plate was significantly weaker than the other two samples, which indicates that the CoP content in these samples follows the order of CoP@a‐CoOx‐5 plate < CoP@a‐CoOx plate ≈ CoP@a‐CoOx‐20. The OER and HER activities of these samples with different CoP contents were then evaluated under the same measurement conditions (Figures S8 and S9, Supporting Information). The CoP@a‐CoOx plate exhibited the most outstanding activity with the smallest overpotential to produce 10 mA cm^−2^ for both OER and HER. The actual content of the crystalline CoP nanoclusters in these heterostructure catalysts was estimated by inductively coupled plasma‐optical emission spectrometry; the results of which were listed in Table S4 (Supporting Information), agreeing well with XPS analysis. In addition, relative to CoP@a‐CoOx‐5 plate and CoP@a‐CoOx‐20 plate, the larger electrochemically active surface area (ECSA) for CoP@a‐CoOx plate was revealed by cyclic voltammetry (CV)‐based electrochemical double layer capacitance measurements (*C*_dl_) (Figure S10, Supporting Information); suggesting the higher amount of active sites in CoP@a‐CoOx plate.

Besides the electrocatalytic activities, the operational durability of the electrocatalyst represents a key factor for practical application. To this end, we performed chronopotentiometric experiments where the OER and HER potentials were monitored as a function of time while 10 mA cm^−2^ OER and −10 mA cm^−2^ HER current densities were applied; the results of which are depicted in Figure 3e. No significant change was observed in both OER and HER potentials profiles during the 24 h testing period, which indicated the remarkable stability of the bifunctional electrocatalytic performance of the CoP@a‐CoOx plate.

**Figure 4:**
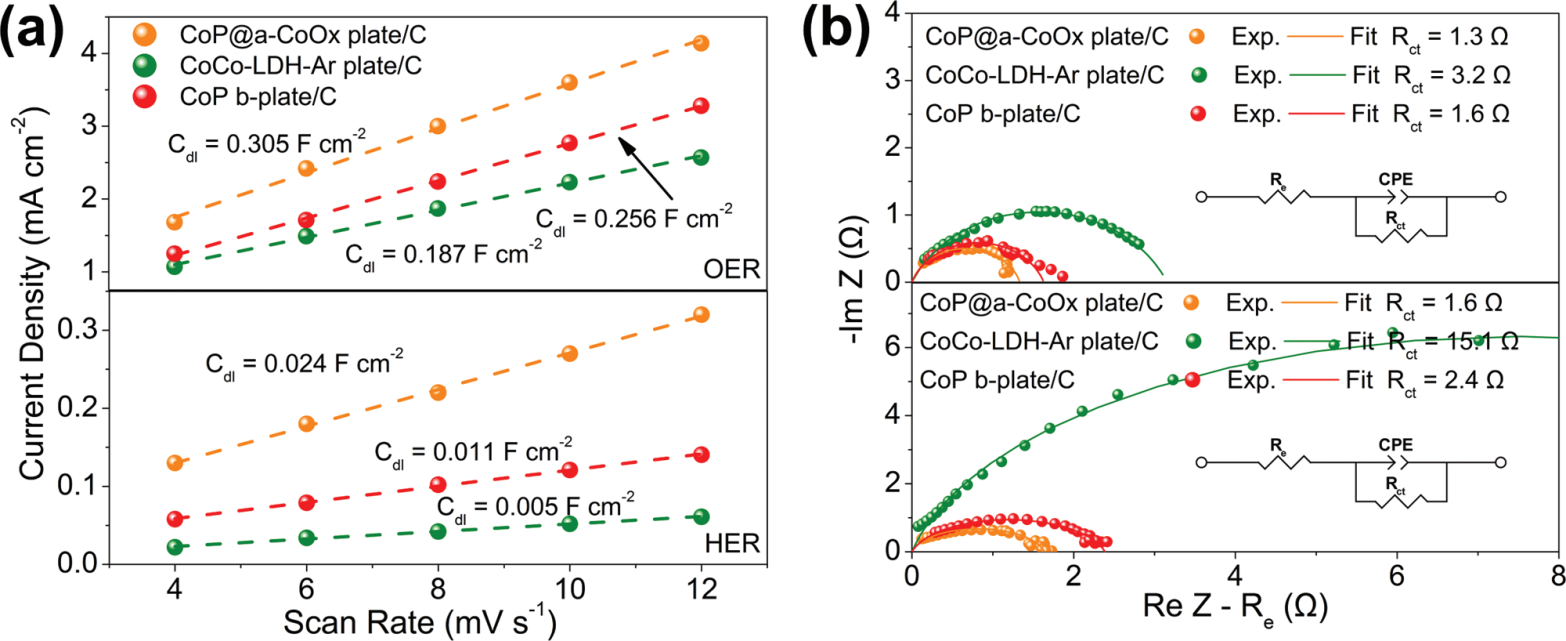
a) Linear fitting of the capacitive currents versus CV scan rates for the CoP@a‐CoOx plate, the CoCo‐LDH‐Ar plate, and the CoP b‐plate samples for OER and HER, and b) Nyquist plots of the CoP@a‐CoOx plate, the CoCo‐LDH‐Ar plate, and the CoP b‐plate catalysts with conductive carbon obtained from EIS measurements at OER potential of 0.6 V versus Ag|AgCl (3.5 m KCl) (above) and HER potential of −1.3 V versus Ag|AgCl (3.5 m KCl) (below).

To determine the reason behind the outstanding OER and HER performances of CoP@a‐CoOx plate, we analyzed several possible contributing factors in sequence. First, it is well‐known that larger ECSA can enhance the electrocatalytic activity.[[qv: 2b,3c]] Our CV‐based electrochemical double layer capacitance measurements (*C*_dl_) results for OER and HER (Figure S11, Supporting Information) revealed that despite its aforementioned lowest BET surface area among all samples, the CoP@a‐CoOx plate indeed exhibited higher ECSA relative to the CoCo‐LDH‐Ar plate and the CoP b‐plate (**Figure** **4**a). This observation suggested that the formation of heterostructure in the CoP@a‐CoOx plate created higher amount of electrocatalytically active sites, which leaded to higher catalytic activity. Second, the electron transfer rate may also play a role towards improving the electrocatalytic activity. We carried out electrochemical impedance spectroscopy (EIS) measurements at 0.6 V and −1.3 V versus Ag|AgCl (3.5 m KCl) to probe the charge transfer properties of OER and HER, respectively; the results of which were displayed in Figure 4b and Figure S12 (Supporting Information). Nyquist plots confirmed the lower charge transfer resistance (*R*_ct_) for the CoP@a‐CoOx plate relative to those for the CoCo‐LDH‐Ar plate and the CoP b‐plate (Figure 4b; Figure S12, Supporting Information). Such result indicated highly efficient electron transfer between the crystalline CoP and the amorphous CoOx nanoclusters. Third, literature reported that amorphous metal oxide sample has higher amount of randomly oriented bonds and higher structural flexibility relative to its crystalline counterpart, which translates to the formation of higher amount of surface unsaturated sites for facile reactant adsorption.^9^ In such metal phosphide, the highly electronegative P atoms can draw electron from metal atoms and act as proton carrier, thus contributing towards enhanced electrocatalytic activity.^10^ More significant is the fact that our XPS results above do indicate strong electronic interaction between the crystalline CoP and amorphous CoOx phases, which led to the favorable modification in the electronic structure and can lower the free energy for adsorption of intermediates, therefore enhancing the catalytic activity.[[qv: 4b,5a,e,f,7]] In the water splitting process, the adsorbed H_2_O molecule electrochemically split and reduced into adsorbed OH^−^ and H atom (H_2_O + e → H_ads_ + OH^−^, Volmer). On the CoP and a‐CoOx nanointerfaces, the OH^−^ could preferentially attach to a a‐CoOx site at the interface due to strong electrostatic affinity to the locally positively charged Co^2+^/Co^3+^ species and more empty d orbitals in Co 2p than that in CoP, while a nearby CoP site would facilitate H adsorption; suggesting synergistic electrocatalytic activity to CoP@a‐CoOx plate. Furthermore, to clarify the roles of each component, i.e., amorphous cobalt oxides or cobalt phosphides, the CoP particles (CoP‐p) were prepared according to the phosphating process of cobalt chloride while amorphous cobalt oxides (a‐CoOx‐p) were prepared following the procedure of the previous report.^11^ The powder XRD patterns of CoP‐p and a‐CoOx‐p confirmed the presence of crystalline CoP phase (JCPDS No. 29‐0497) and amorphous CoOx structure, respectively (Figure S13a, Supporting Information). Shown in Figure S13b,c of the Supporting Information were the OER and HER polarization curves, respectively, for CoP@a‐CoOx plate, CoP‐p, a‐CoOx‐p, and the mixture of CoP‐p and a‐CoOx‐p. (The content of CoP in the mixture is similar to that in CoP@a‐CoOx plate.) Upon comparing CoP‐p with a‐CoOx‐p, it becomes clear that CoP was the dominant active center while CoOx provided marginal contribution for both OER and HER. Notably, the mixture of CoP‐p and a‐CoOx‐p showed much lower activities than CoP‐p, which was likely due to the reduction of the dominant active CoP for the mixture under the same catalyst loading on the electrode. However, both OER and HER activities for CoP@a‐CoOx plate were significantly higher than those for the three other samples. Thus, these further confirmed the synergistic effect of CoP nanoclusters and amorphous CoOx nanoplates in the CoP@a‐CoOx plate sample. We postulated the collective contribution from these factors as the reason behind the observed significant enhancement in the OER and HER activities of the CoP@a‐CoOx plate.

**Figure 5:**
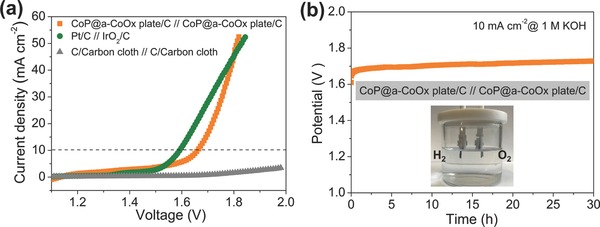
a) Polarization curves of the CoP@a‐CoOx plate/C||CoP@a‐CoOx plate/C system, the Pt/C(−)||IrO_2_/C(+) system, and the C/Carbon cloth||C/Carbon cloth system for overall electrochemical water splitting in a 1 m KOH solution with an individual electrode mass loading of 5 mg cm^−2^; obtained using a 5 mV s^−1^ scan rate. b) Chronopotentiometric (potential versus time) plot of electrochemical water splitting using the CoP@a‐CoOx plate/C||CoP@a‐CoOx plate/C at a constant current density of 10 mA cm^−2^ in a 1 m KOH solution. Inset: a representative photograph of water‐splitting device.

We then assessed bifunctional electrocatalytic activity of the CoP@a‐CoOx plate for water electrolysis in two‐electrode alkaline electrolyzer. The CoP@a‐CoOx plate was loaded on carbon cloth substrate as both the anode and the cathode components with mass loading of 5 mg cm^−2^. As a baseline comparison, Pt/C||IrO_2_/C and C/carbon cloth||C/carbon cloth electrodes systems were also constructed and tested. **Figure** **5**a showed the linear polarization curves for electrochemical water splitting for these three systems. The coupling of the OER and HER benchmarks in single system clearly manifested into the lowest potential of ≈1.590 V to sustain operation at 10 mA cm^−2^ current density (Figure 5a). Still, the CoP@a‐CoOx plate electrodes displayed a closely approaching electrolysis performance with a relatively low potential of ≈1.660 V at similar current density (Figure 5a). Such performance was comparable or even higher than those of the reported best‐performing nonprecious metal catalysts for water electrolysis to date (Table S5, Supporting Information).[[qv: 4b,12]] Despite its more positive onset potential for water electrolysis, the CoP@a‐CoOx plate/C||CoP@a‐CoOx plate/C system displayed more rapid increase in current density with an increase in potential (Figure 5a). As a result, its performance reached that of the Pt/C||IrO_2_/C system at ≈1.80 V versus RHE (Figure 5a). The C/carbon cloth||C/carbon cloth system, on the other hand, displayed almost negligible water electrolysis activity between 1 and 2 V versus RHE (Figure 5a). It is also noteworthy that higher loading for CoP@a‐CoOx plate/C (8 mg cm^−2^) resulted in a higher performance for overall water splitting and our developed catalysts also displayed higher water‐spilitting activity than that of the benchmark Pt/C||IrO_2_/C system with the same loading in a high current density range, approximately above 0.1 A cm^−2^ (Figure S14, Supporting Information). The long term operational stability of the CoP@a‐CoOx plate electrode system was furthermore evaluated by chronopotentiometric test, i.e., potential monitoring while a current density of 10 mA cm^−2^ was applied; the result of which was shown in Figure 5b. Only slight increase of potential over 30 h test period indicated the durability of the CoP@a‐CoOx plate/C||CoP@a‐CoOx plate/C system during alkaline water electrolysis.

## 3 Conclusion

In summary, we have reported the formation of heterostructure in the CoP@a‐CoOx plate where the crystalline CoP nanoclusters were embedded within the amorphous CoOx matrix, which displayed very high OER activity in addition to high HER activity in an alkaline media. Such outstanding bifunctional activities were also accompanied by high operational stability; suggesting its potential for use in practical alkaline water electrolysis device. The CoP@a‐CoOx plate was synthesized via combined solvothermal and low temperature phosphidation route. We attributed the significant enhancement in OER and HER catalytic activities of the CoP@a‐CoOx plate over those of the CoCo‐LDH‐Ar plate and the CoP b‐plate to the presence of synergy between the two phases as created by strong interactions between them in such heterostructure, resulting in increased ECSA, enhanced charge transfer rate, and more importantly, the modified electronic configuration. We also demonstrated an efficient and stable alkaline water electrolysis operation using the CoP@a‐CoOx plate as both the cathode and anode components. In addition to these superior electrocatalytic properties, its low cost, natural abundance, and environmental compatibility increase its attractiveness for use in large scale industrial electrolysis application.

## 4 Experimental Section

*Catalysts Synthesis*: Synthesis of CoCo‐LDH plate: The CoCo‐LDH plate was prepared via a solvothermal route according to a previous report.^13^ In brief, 0.5 g of cobaltous acetate was added to 36 mL of EG. Following dissolution by ultrasonication, the solution mixture was heated at 200 °C for 5 h under continuous stirring. The solution became turbid at the end of the reaction due to the gradual formation of CoCo layered double hydroxides precursors (CoCo‐LDH plate). The mixture was then cooled down naturally to room temperature. The pink precipitate was recovered by suction filtration, rinsed with de‐ionized (DI) water and ethanol several times, and finally dried at 60 °C for overnight.

*Synthesis of CoP@a‐CoOx Plate*: To synthesize the CoP@a‐CoOx plate, the CoCo‐LDH plate was phosphidated using the phosphorus vapor from sodium hypophosphite (NaH_2_PO_2_). In brief, CoCo‐LDH plate was introduced into a tube reactor. NaH_2_PO_2_ was also introduced in separate upstream location in a tube reactor. The mass ratio of NaH_2_PO_2_ and CoCo‐LDH plate is 10:1. Argon (Ar) was then flowed through the tube for 30 min before heating. The tube was then heated inside tubular furnace to 300 °C using a ramping rate of 1 °C min^−1^ in a static Ar atmosphere. The heating at 300 °C was maintained for 1 h.

*Synthesis of CoP b‐Plate and CoCo‐LDH‐Ar Plate*: As a baseline comparison, some of the CoP@a‐CoOx plate was subjected to acid etching (3 m HCl) for 6 h to disintegrate the CoP plate, which is denoted as the CoP b‐plate. The CoCo‐LDH‐Ar plate was also prepared using a same procedure with the CoP@a‐CoOx plate in the absence of the NaH_2_PO_2_.

*Materials Characterization*: The phase structures of the samples were evaluated using powder XRD measurements on a Rigaku Smartlab diffractometer using filtered Cu‐Kα radiation (λ = 1.5418 Å) in 2θ range of 10°–90°. The morphologies of the samples were observed by a HITACHI‐S4800 field‐emission SEM and an FEI Tecnai G2T20 transmission electron microscope. Additionally, the corresponding STEM‐EDX line scan and element mapping were taken on an FEI Tecnai G2 F30 STWIN field‐emission transmission electron microscope equipped with an EDX analyzer at 200 kV. XPS measurement was conducted to probe the chemical compositions and surface element states on a PHI5000 VersaProbe spectrometer equipped with an Al‐Kα X‐ray source and the data were fitted by the software package XPSPEAK. The specific surface areas and pore size distributions were determined from the N_2_ adsorption–desorption isotherms using the BET and Barrett–Joyner–Halenda methods.

*Electrode Preparation and Electrochemical Measurements*: All electrochemical measurements were carried out in room temperature in a standard three‐electrode electrochemical cell with the carbon cloth as the working electrode substrate, a graphite rod (HER) or a platinum wire (OER) as the counter electrode, and an Ag|AgCl (3.5 m KCl) as the reference electrode; the operation of which was controlled by a CHI 760E bi‐potentiostat. All tests were performed in a 1 m KOH electrolyte (pH ≈ 13.7). For the preparation of a working electrode, 10 mg of each catalyst sample and 10 mg conductive carbon were dispersed in a mixture of 100 µL of 5 wt% Nafion solution and 1 mL of ethanol by mild sonication for at least 1 h to form the homogeneous working electrode ink. Then, a 83 µL of ink aliquot was pipetted onto the carbon cloth substrate with 0.5 cm^2^ geometric surface area; leading to an approximate catalyst loading of 1.5 mg_catalyst_ cm^−2^ (3 mg_total_ cm^−2^). Commercial IrO_2_ (99.9%, Aladdin Industrial Corporation, 0.6 m^2^ g^−1^) and Pt/C (20 wt%, Johnson‐Matthey, 138.8 m^2^ g^−1^) catalysts were purchased and used as received. Electrodes containing commercial IrO_2_ were obtained in similar manner. Besides, electrodes containing commercial Pt/C catalyst were prepared using the similar procedure except for that 20 mg total sample (10 mg of each catalyst sample and 10 mg conductive carbon) was substituted by 10 mg Pt/C.

For ≈30 min prior to the start of each test till the end of test, the electrolyte was continuously bubbled with O_2_ or Ar (O_2_ for OER tests and Ar for HER tests). CV measurements were first carried out for pretreatment at different potential regions versus Ag|AgCl for OER and HER tests. Then, the OER polarization curves were recorded by using LSV at a 5 mV s^−1^ scan rate from 0.2 to 1.0 V versus Ag|AgCl (3.5 m KCl) and HER polarization curves was recorded from −0.8 to −1.6 V versus Ag|AgCl (3.5 m KCl). All potentials were calibrated against and converted to RHE after iR‐corrected: *E*_RHE_ =*E*_Ag/AgCl_ +0.197 + 0.059 × pH. The polarization curves were replotted as overpotential (η) versus the logarithm of current density (log |j|) to obtain Tafel plots. The electrochemical double‐layer capacitance, Cdl, was calculated from CV curves, which is recorded in a potential range with no Faradaic current at different scan rates from 20 to 120 mV s^−1^. EIS measurements were performed over a frequency range from 100 kHz to 0.1 Hz at 0.6 V versus Ag|AgCl (3.5 m KCl) for OER and −1.3 V versus Ag|AgCl (3.5 m KCl) for HER under the influence of an AC bias potential of 5 mV. To achieve clear comparison of electrode polarization resistances, the electrolyte resistance (*R*_e_) obtained at the high frequency was subtracted from each spectrum. For simplicity of the model, the Warburg impedance was neglected, which leads to the large error at low frequency regime. All Nyquist plots exhibit one semicircular response. The Randles circuit, which consists of an electrolyte resistance (*R*_e_), a charge transfer resistance (*R*_ct_) and a constant phase element (CPE), is used. CPE is used to account for the nonideality in the electrode that causes a frequency dispersion in the capacitance response. The long‐term stability was tested at a constant current density of 10 mA cm^−2^ for 10 h. The overall water splitting tests were conducted in a home‐made two‐electrode system with catalysts loaded on carbon cloth (mass loading of 5 mg cm^−2^). The polarization curves were obtained with a 5 mV s^−1^ scan rate and were further *iR*‐corrected.

## Conflict of Interest

The authors declare no conflict of interest.

## Supporting information

Supplementary
