# Supplementary material for: Bifunctionality from Synergy: CoP Nanoparticles Embedded in Amorphous CoOx Nanoplates with Heterostructures for Highly Efficient Water Electrolysis
Source: Adv Sci (Weinh). 2018 Jul 13;5(9):1800514. doi: 10.1002/advs.201800514 (PMC6145296; doi:10.1002/advs.201800514)
Supplement: Supplementary file 1 — Supplementary [file ADVS-5-1800514-s001.pdf]

Copyright WILEY-VCH Verlag GmbH & Co. KGaA, 69469 Weinheim, Germany, 2018.

ADVANCED SCIENCE
Open Access

Supporting Information

for *Adv. Sci.*, DOI: 10.1002/advs. 201800514

Bifunctionality from Synergy: CoP Nanoparticles Embedded in Amorphous CoOx Nanoplates with Heterostructures for Highly Efficient Water Electrolysis

Jie Yu, Yijun Zhong, Xinhao Wu, Jaka Sunarso, Meng Ni, Wei Zhou, and Zongping Shao**

Copyright WILEY-VCH Verlag GmbH & Co. KGaA, 69469 Weinheim, Germany, 2016.

Supporting Information

Bifunctionality from synergy: CoP nanoparticles embedded in amorphous CoOx nanoplates with heterostructures for highly efficient water electrolysis

Jie Yu, Yijun Zhong, Xinhao Wu, Jaka Sunarso, Meng Ni, Wei Zhou and Zongping Shao**

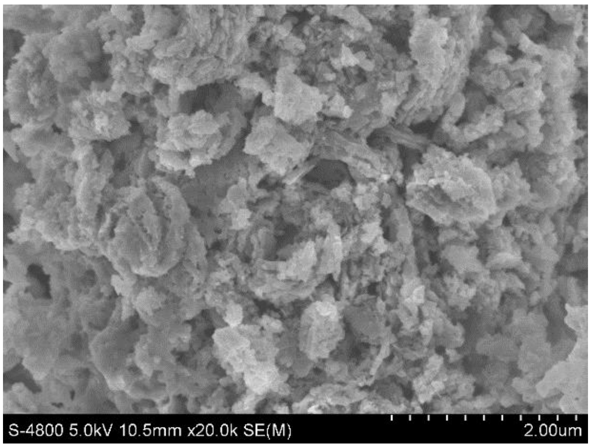

Figure S1. SEM image of the CoP b-plate that features disintegrated platelets.

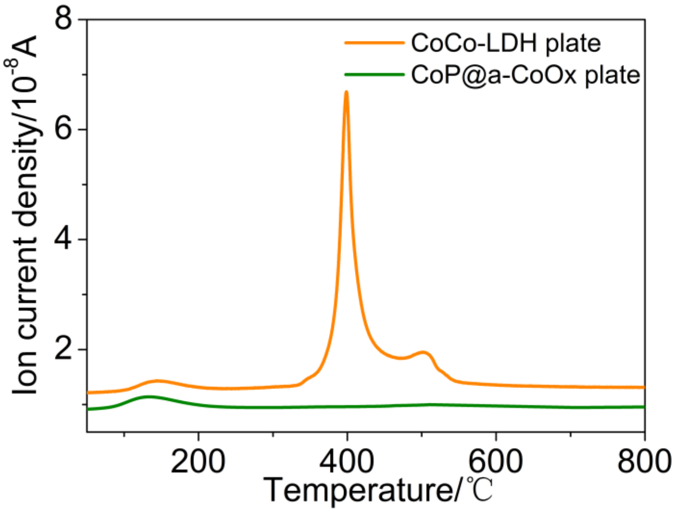

Figure S2. The qualitative determination of gas components (H_2O) of CoCo-LDH plate and CoP@a-CoOx plate during the thermal decomposition process.

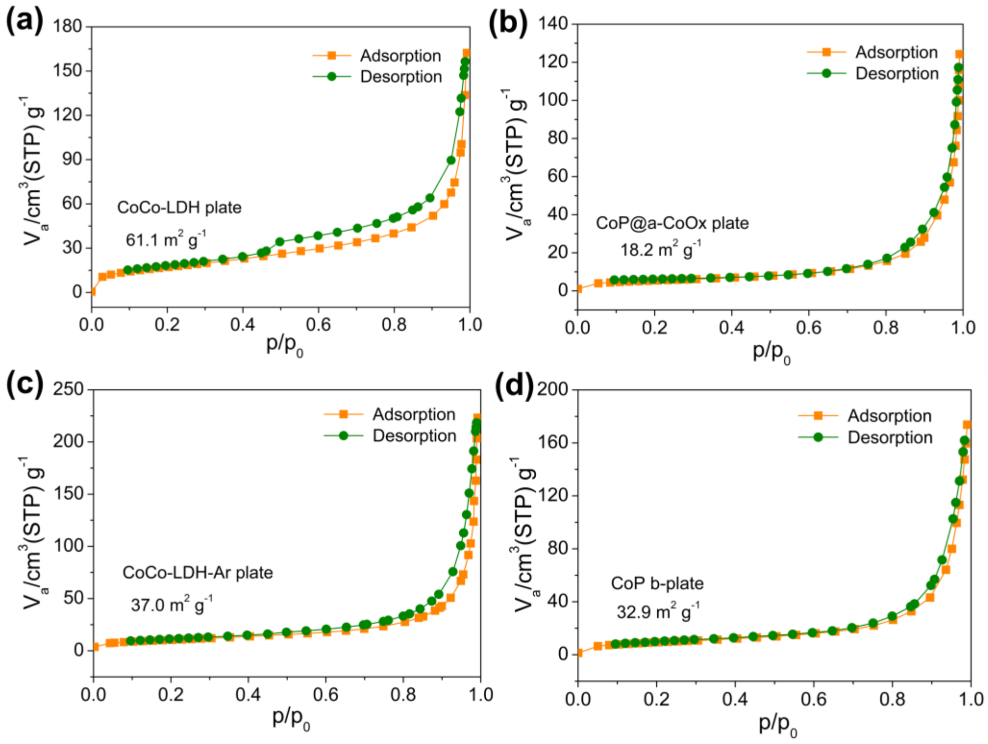

Figure S3. Nitrogen adsorption-desorption isotherms of **a)** the CoCo-LDH plate; **b)** the CoP@a-CoOx plate; **c)** the CoCo-LDH-Ar plate; and **d)** the CoP b-plate.

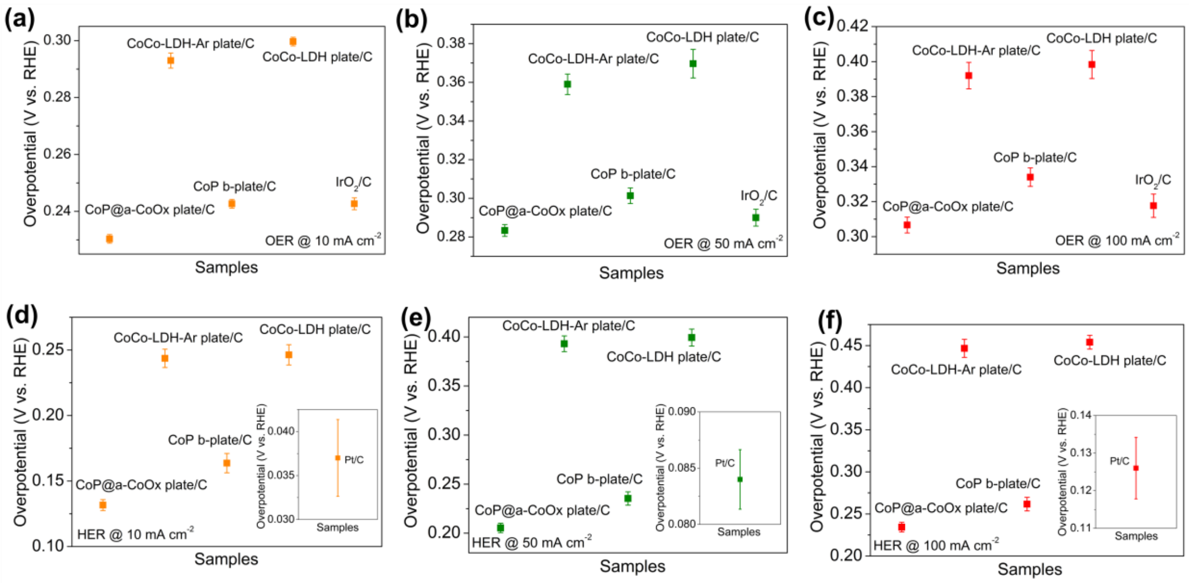

Figure S4. Overpotentials at the current densities of **a, d)** 10; **b, e)** 50; and **c, f)**100 mA cm^{-2} for both **a-c)** OER and **d-f)** HER among different catalysts, respectively. Error bars represent standard deviations from at least three independent measurements.

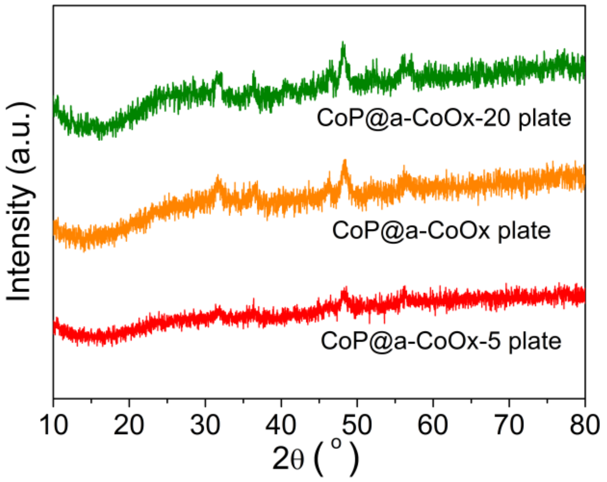

Figure S5. Powder XRD patterns of the CoP@a-CoOx-5 plate, the CoP@a-CoOx plate, and the CoP@a-CoOx-20 plate samples.

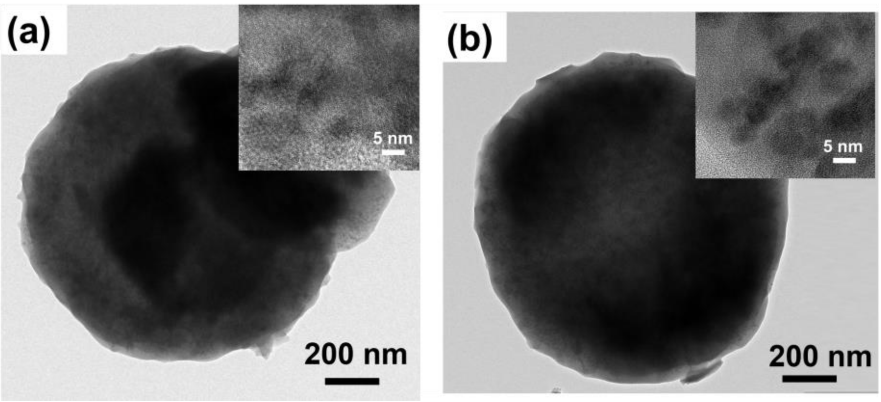

Figure S6. TEM images of **a)** CoP@a-CoOx-5 plate; and **b)** CoP@a-CoOx-20 plate.

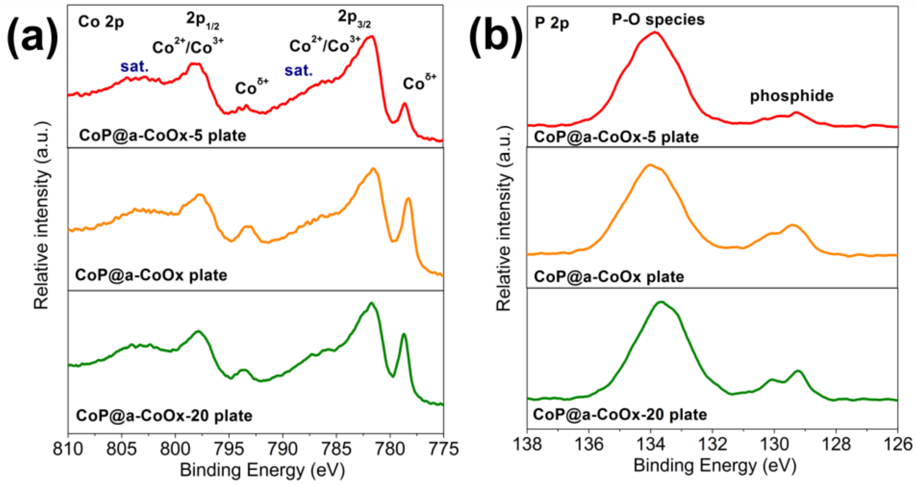

Figure S7. High resolution XPS spectra of **a)** Co 2p; and **b)** P 2p for CoP@a-CoOx-5 plate, CoP@a-CoOx plate, and CoP@a-CoOx-20 plate.

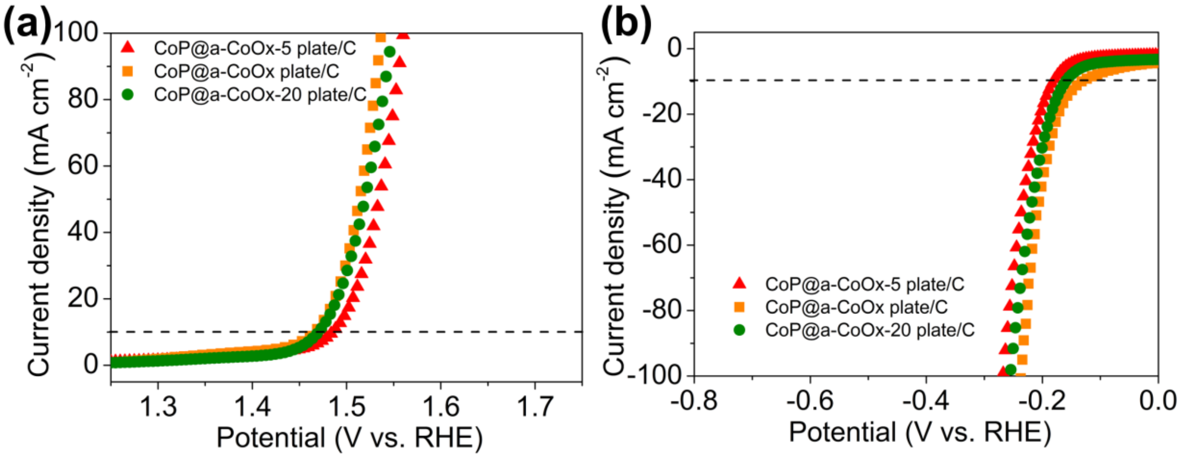

Figure S8. a) OER polarization curves of the CoP@a-CoOx-5 plate, the CoP@a-CoOx plate, and the CoP@a-CoOx-20 plate samples loaded on the carbon cloth substrate in an O_2-saturated 1 M KOH solution; obtained using a 5 mV s^{-1} scan rate; **b)** HER polarization curves of the CoP@a-CoOx-5 plate, the CoP@a-CoOx plate, and the CoP@a-CoOx-20 plate samples loaded on the carbon cloth substrate in an Ar-saturated 1 M KOH solution; obtained using a 5 mV s^{-1} scan rate.

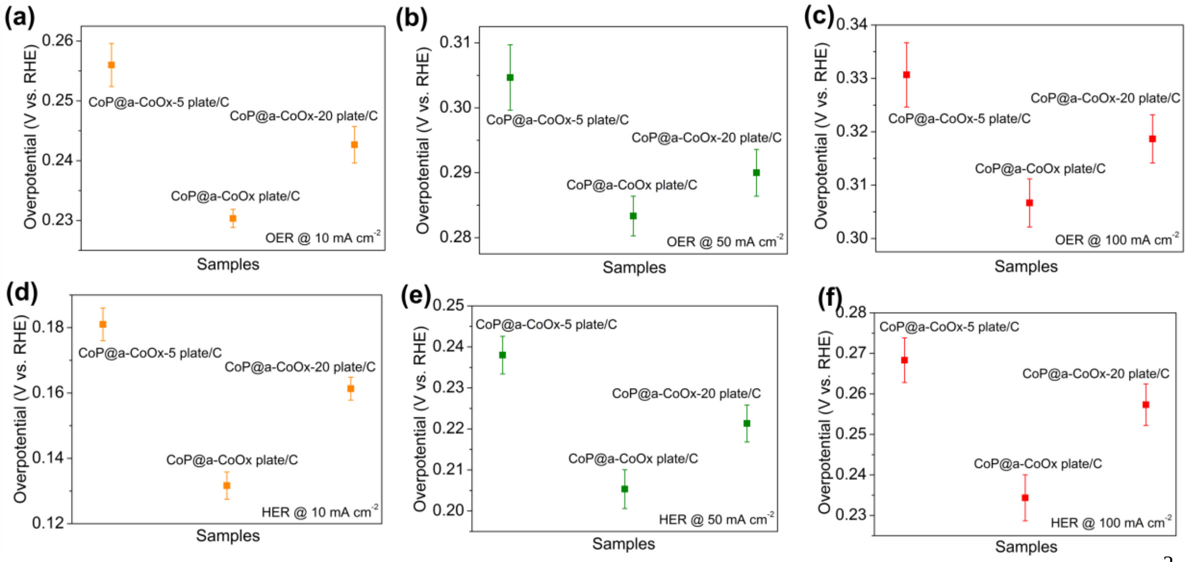

Figure S9. Overpotentials at the current densities of **a, d)** 10; **b, e)** 50; and **c, f)**100 mA cm^{-2} for both **a-c)** OER and **d-f)** HER among the CoP@a-CoOx-5 plate, the CoP@a-CoOx plate, and the CoP@a-CoOx-20 plate samples, respectively. Error bars represent standard deviations from at least three independent measurements.

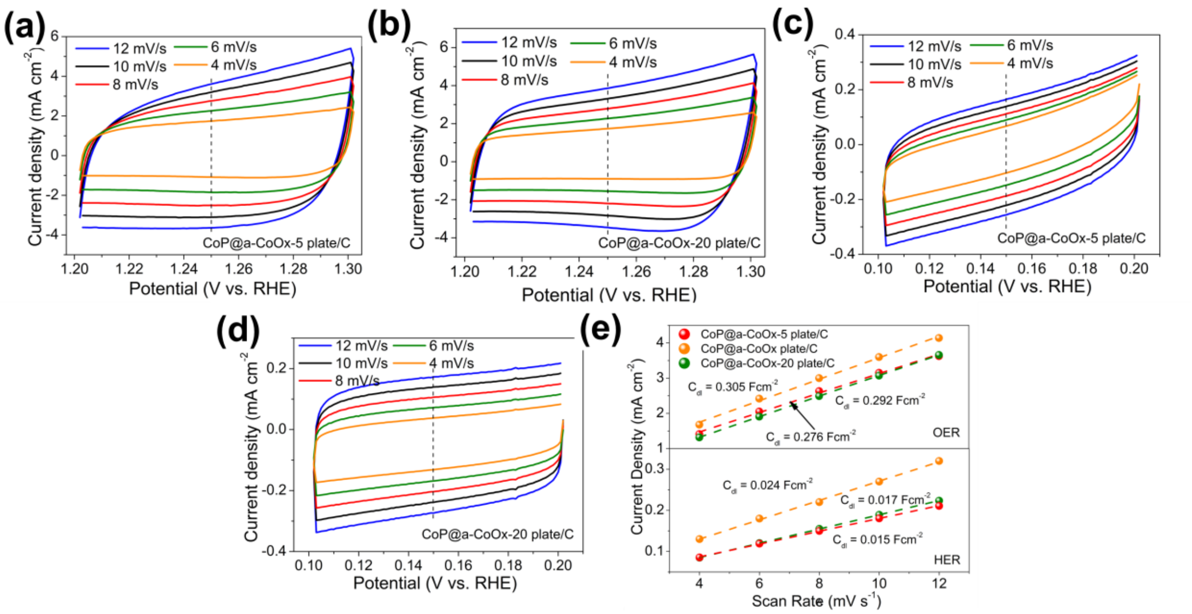

Figure S10. Cyclic voltammetry profiles for **a) and c)** the CoP@a-CoOx-5 plate/C; and **b) and d)** the CoP@a-CoOx-20 plate/C at different scan rates (i.e., 4-12 mV s^{-1}) in a 1 M KOH solution; and **e)** linear fitting of the capacitive currents versus CV scan rates for the CoP@a-CoOx-5 plate/C, CoP@a-CoOx plate/C, and CoP@a-CoOx-20 plate/C samples for OER and HER.

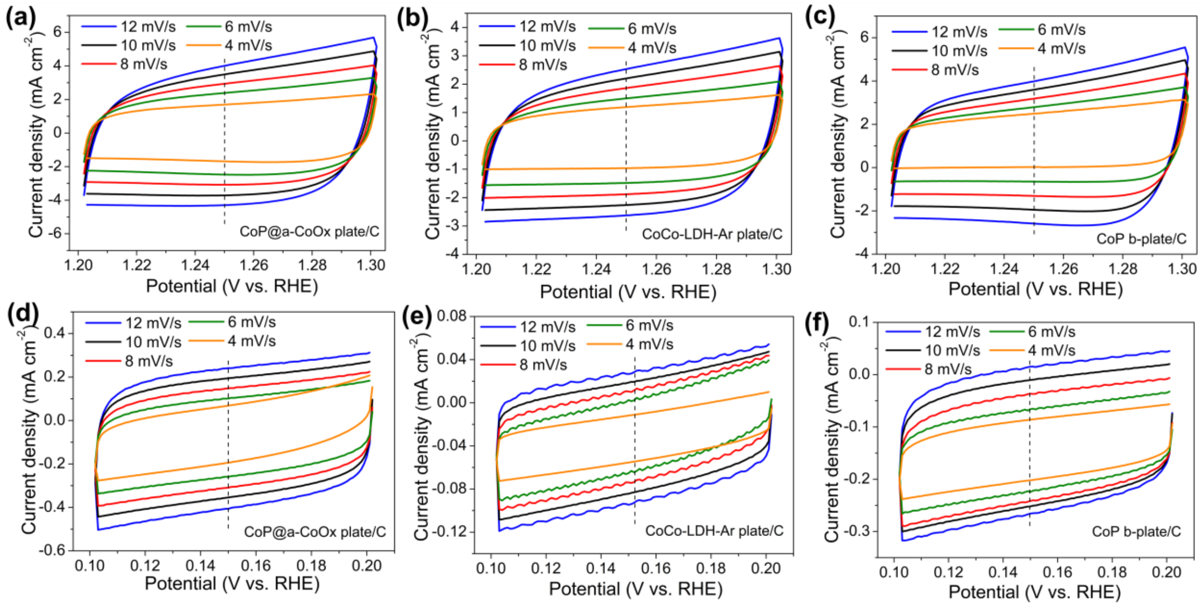

Figure S11. Cyclic voltammetry profiles for **a) and d)** the CoP@a-CoOx plate/C; **b) and e)** the CoCo-LDH-Ar plate/C; and **c) and f)** the CoP b-plate/C at different scan rates (i.e., 4-12 mV s^{-1}) in a 1 M KOH solution; **a), b), and c)** OER; and **d), e), and f)** HER estimated ECSAs.

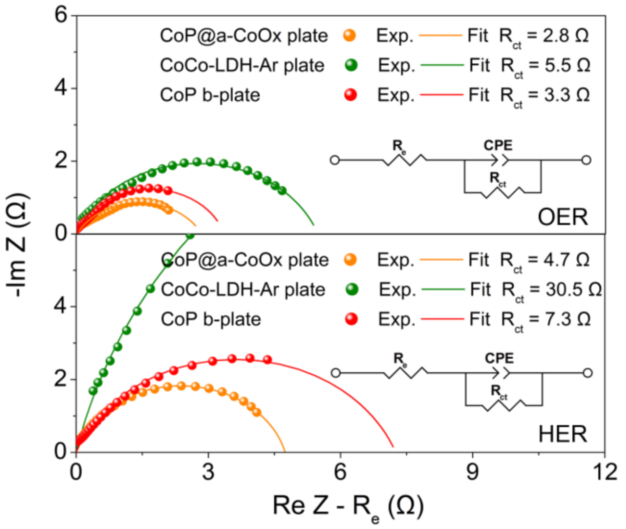

Figure S12. Nyquist plots of the CoP@a-CoOx plate, the CoCo-LDH-Ar plate, and the CoP b-plate catalysts obtained from EIS measurements at OER potential of 0.6 V vs. Ag|AgCl (3.5 M KCl) and HER potential of -1.3 V vs. Ag|AgCl (3.5 M KCl).

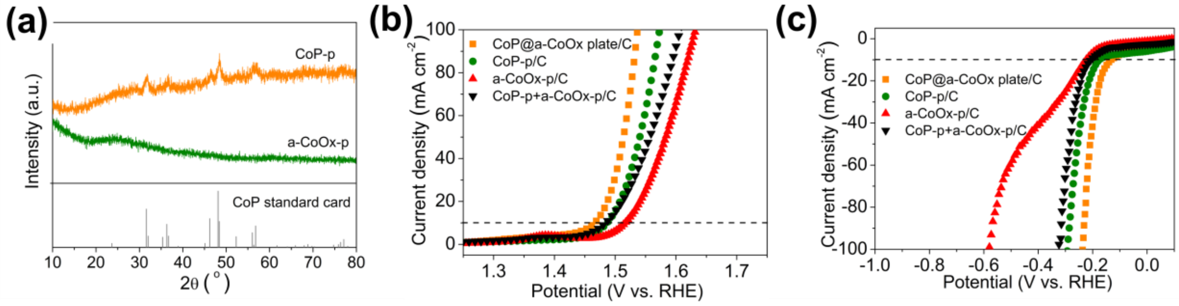

Figure S13. a) Powder XRD patterns of the CoP-p, and the a-CoOx-p samples; **b)** OER polarization curves; and **c)** HER polarization curves of CoP@a-CoOx plate, CoP-p, a-CoO_x-p, and the mixture of CoP-p and a-CoO_x-p.

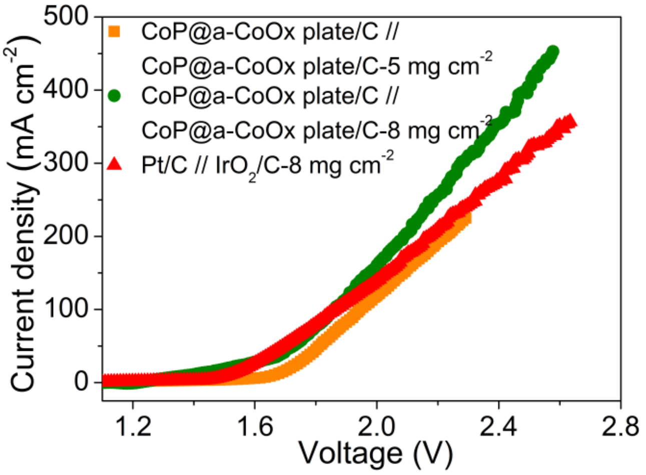

Figure S14. Polarization curves of the CoP@a-CoOx plate/C || CoP@a-CoOx plate/C-5 mg cm^{-2} system, the CoP@a-CoOx plate/C || CoP@a-CoOx plate/C-8 mg cm^{-2} system, and the Pt/C(−) || IrO_2/C(+)-8 mg cm^{-2} system for overall electrochemical water splitting in a 1 M KOH solution.

Table S1. Specific surface areas for different samples.

Sample	S_{BET}[a] (m^2 g^{-1})
CoCo-LDH plate	61.1
CoP@a-CoOx plate	18.2
CoCo-LDH-Ar plate	37.0
CoP b-plate	32.9
CoP@a-CoOx-5 plate	21.4
CoP@a-CoOx-20 plate	17.8

[a] S_{BET}: specific surface area from BET method.

Table S2. Comparison of selected non-precious OER electrocatalysts in an alkaline medium.

Catalyst	Electrolyte	Loading (mg cm^{-2})	Substrate	η_{10} (V)	Tafel slope (mV dec^{-1})	References
**CoP@a-CoOx plate**	**1 M KOH**	**1.5**	**Carbon cloth**	**0.23**	**67**	**This work**
**IrO_2**	**1 M KOH**	**1.5**	**Carbon cloth**	**0.24**	**59**	**This work**
CoP MNA	1 M KOH	6.2	Ni foam	0.29	65	*Adv. Funct. Mater.* **2015**, *25*, 7337.
Co_4N	1 M KOH	0.82	Carbon cloth	0.26	44	*Angew. Chem. Int. Ed.* **2015**, *54*, 14710.
NiCoP/CC	1 M KOH	2	Carbon cloth	0.24	64	*ACS Catal.* **2017**, *7*, 4131.
$Co_{1.04}Fe_{0.96}P$	1 M KOH	1	Self-supported	0.27	30	*Energy Environ. Sci.* **2016**, *9*, 2257.
a-NiFe-OH/NiFeP/NF	1 M KOH	1.8	Ni foam	0.20	39	*ACS Energy Lett.* **2017**, *2*, 1035.
CoN-1min	1 M KOH	1.5	Ni foam	0.29	70	*Angew.Chem. Int. Ed.* **2016**, *55*,8670.
Fe-Ni oxides	1 M KOH	0.5	Carbon paper	>0.37	51	*ACS Catal.* **2012**, *2*, 1793.
Co-P film	1 M KOH	2.71	Cu foil	0.34	47	*Angew. Chem. Int. Ed.* **2015**, *54*, 6251.
CoCo LDH	1 M KOH	1	Ni foam	0.39	59	*Nat. Commun.* **2014**, *5*, 4477.
$Zn_{0.75}Co_{2.25}O_4$ nanowire array	1 M KOH	1	Ti foil	0.32	51	*Chem. Mater.* **2014**, *26*, 1889.

Table S3. Comparison of selected non-precious HER electrocatalysts in an alkaline medium.

Catalyst	Electrolyte	Loading	Substrate	η_{10}	Tafel slope	References

		(mg cm^{-2})		(V)	(mV dec^{-1})	
**CoP@a-CoOx plate**	**1 M KOH**	**1.5**	**Carbon cloth**	**0.13**	**89**	**This work**
CoP/CC	1 M KOH	0.92	Carbon cloth	0.21	129	*J. Am. Chem. Soc.* **2014**, *136*, 7587.
Ni_2P	1 M KOH	176.9	Ti foil	0.07	118	*Energy Environ. Sci.* **2015**, *8*, 1027.
NiO/Ni-CNT	1 M KOH	8	Ni foam	<0.1	51	*Nat. Commun.* **2014**, *5*, 4695.
WP NAs/CC	1 M KOH	2	Carbon cloth	0.15	102	*ACS Appl. Mater. Interfaces* **2014**, *6*, 21874.
FeP NAs/CC	1 M KOH	1.5	Carbon cloth	0.22	146	*ACS Catal.* **2014**, *4*, 4065.
HNDCM-Co/CoP	1 M KOH	42.8mg$_{Co}$/g$_{total}$	Self-supported	0.135	64	*ACS Nano* **2017**, *11*, 4358.
$NiCo_2S_4$ NW/NF	1 M KOH	---	Ni foam	0.21	59	*Adv. Funct. Mater.* **2016**, *26*, 4661.
CP@Ni-P	1 M KOH	25.8	Carbon paper	0.12	85	*Adv. Funct. Mater.* **2016**, *26*, 4067.

Table S4. The actual content of the crystalline CoP nanoclusters in these heterostructure catalysts.

Sample	The content of CoP (wt %)
CoP@a-CoOx-5 plate	13.4
CoP@a-CoOx plate	37.2
CoP@a-CoOx-20 plate	42.3

To obtain the actual content of the crystalline CoP nanoclusters in these heterostructure catalysts, inductively coupled plasma-optical emission spectrometry (ICP-OES) measurements were performed. Specifically, 20 mg of a catalyst was absolutely dissolved into a certain amount of concentrated nitric acid, and then it was diluted to 1 L with the addition of deionized water. The amount of cobalt and phosphorus in this solution was quantified using ICP-OES. For the CoP@a-CoOx plate sample, the amount of cobalt and phosphorus was 14.03 mg/L (14.03 ppm) and 2.56 mg/L (2.56 ppm) in the obtained solution, respectively. Thus, the actual content of the crystalline CoP nanoclusters in this hybrid can be calculated as follows: $2.56/30.97 \times (58.93+30.97)/20 = 37.2$ %.

Table S5. Comparison of the performance of the water-splitting catalysts in a 1.0 M KOH solution.

Anode	Cathode	Substrate	Loading (mg cm^{-2})	η_{10} (V)	References
**CoP@a-CoOx plate**	**CoP@a-CoOx plate**	**Carbon cloth**	**5**	**1.66**	**This work**

**IrO_2/C**	**Pt/C**	**Carbon cloth**	**5**	**1.59**	**This work**
NiFe LDHs	NiFe LDHs	Ni foam	---	1.70	*Science* **2014**, *345*, 1593.
$Co_{0.85}$Se/NiFe-LDH	$Co_{0.85}$Se/NiFe-LDH	Exfoliated graphene foil	4	1.67	*Energy Environ. Sci.* **2016**, *9*, 478.
High-index faceted Ni_3S_2 nanosheet	High-index faceted Ni_3S_2 nanosheet	Ni foam	1.6	>1.7	*J. Am. Chem. Soc.* **2015**, *137*, 14023.
Co_2B-500	Co_2B-500/NG	Carbon cloth	5	1.81	*Adv. Energy Mater.* **2016**, *6*, 1502313.
Co-P	Co-P	Ni foam	2.6	~ 1.64	*Angew. Chem.* **2015**, *127*, 6349.
SNCF-NR	SNCF-NR	Ni foam	3	1.68	*Adv. Energy Mater.* **2017**, *7*, 1602122.
Co/CoP-5	Co/CoP-5	Ni foam	5	1.45	*Adv. Energy Mater.* **2017**, *7*, 1602355.
Ni/NiP	Ni/NiP	Ni foam	10.58	1.61	*Adv. Funct. Mater.* **2016**, *26*, 3314.
NiSe/NF	NiSe/NF	Ni foam	2.8	1.63	*Angew. Chem.* **2015**, *127*, 9483.
